# Artificial Intelligence and Declined Guilt: Retailing Morality Comparison Between Human and AI

**DOI:** 10.1007/s10551-022-05056-7

**Published:** 2022-02-12

**Authors:** Marilyn Giroux, Jungkeun Kim, Jacob C. Lee, Jongwon Park

**Affiliations:** 1 Department of Marketing, Auckland University of Technology, 120 Mayoral Drive, Auckland, 1010 New Zealand; 2 Dongguk Business School, Dongguk University, 30 Pildong-ro 1-gil, Seoul, Korea; 3 Department of Business Administration, Korea University, 1 Anam-Dong, Sungbuk-Gu, Seoul, Korea

**Keywords:** Artificial intelligence, Self-service technologies, AI technology, AI morality, Human–machine interaction, Moral judgments, Machine versus AI, Human versus AI

## Abstract

Several technological developments, such as self-service technologies and artificial intelligence (AI), are disrupting the retailing industry by changing consumption and purchase habits and the overall retail experience. Although AI represents extraordinary opportunities for businesses, companies must avoid the dangers and risks associated with the adoption of such systems. Integrating perspectives from emerging research on AI, morality of machines, and norm activation, we examine how individuals morally behave toward AI agents and self-service machines. Across three studies, we demonstrate that consumers’ moral concerns and behaviors differ when interacting with technologies versus humans. We show that moral intention (intention to report an error) is less likely to emerge for AI checkout and self-checkout machines compared with human checkout. In addition, moral intention decreases as people consider the machine less humanlike. We further document that the decline in morality is caused by less guilt displayed toward new technologies. The non-human nature of the interaction evokes a decreased feeling of guilt and ultimately reduces moral behavior. These findings offer insights into how technological developments influence consumer behaviors and provide guidance for businesses and retailers in understanding moral intentions related to the different types of interactions in a shopping environment.

## Introduction

Several technological developments, such as the Internet, self-service technologies, and artificial intelligence (AI), have entirely changed the face of retailing, altering purchase and consumption patterns and interactions within the retail environment (Grewal et al., 2018; Guha et al., 2021; Kumar et al., 2020; Rafaeli et al., 2017). Those innovations become an essential part of businesses and continue to shift how marketing strategies are implemented in terms of customer service and managing relationships and sales. Those systems are completely shaking up the retail landscape, with global corporations (e.g., Alibaba, Amazon, McDonald’s, and Walmart) investing massively to project themselves in the new retailing experience (Kaplan, 2020; Kats, 2020). Several opportunities, such as self-service checkouts and recommendation systems-based data analysis, can result in considerable gains for businesses and long-term cost savings and contribute to the overall consumer experience (Davenport et al., 2020; Grewal et al., 2018; Kim, 2017; Oh et al., 2013).

As a result of their increased availability, retailers and service companies are using those technologies to predict consumers’ preferences and create a fast, smooth, and convenient experience for customers. Due to the numerous benefits attached to those technologies, such as reducing operating costs and increased ability and efficacy for consumers (Bulmer et al., 2018), businesses have focused on developing means to stimulate their adoption. Past literature has mainly investigated technological issues related to AI and consumers’ reactions to these technological advancements. Indeed, despite their growing usage, researchers have documented a certain resistance to the adoption of AI not only due to regulation and privacy issues but also due to their inaccuracy to make inferences and predictions (Dietvorst et al., 2015; Longoni et al., 2019) and the inauthenticity of those predictions (Jago, 2019). Individuals can also perceive more risk and experience discomfort when interacting with automated services and robots (Huang & Rust, 2018). Even if AI usage presents positive outcomes (e.g., cost savings) for businesses, negative aspects and undesirable behaviors from consumers such as augmentation and normalization of consumer theft and shoplifting have also been observed (Dimoff, 2020; Taylor, 2016). Past literature has investigated how individuals respond morally to actions and decisions by machines and AI agents. However, more investigation of consumers’ moral decisions toward those technologies is needed.

Given the rising usage of technologies in the retailing environment, more research is warranted to identify how consumers are reacting toward those advancements to increase their successful implementation and uncover the underlying processes that explain how those technologies impact consumer behavior. This research examines the moral aspects of consumption and shopping in retail environments embracing diverse technologies. How people morally respond to available technologies and the factors explaining those reactions must be explored. Although prior research has investigated how individuals morally perceive and react to behaviors from machines and robots (Awad et al., 2019; Bigman & Gray, 2018; Gamez et al., 2020) or social media platforms, such as WeChat (He & Tan, 2021), research on consumers’ moral reactions toward those technologies is limited.

Given the substantial development in the area of AI and automated services, this study investigates how individuals morally behave toward AI agents and self-service machines along with recent interests regarding morality issues with new technology (e.g., Brusoni & Vaccaro, 2017; Johnson, 2015; Martin & Freeman, 2004; Martin et al., 2019). Specifically, we investigate whether consumers’ moral concerns and behaviors toward machines and their reactions toward their human counterparts vary. We show that moral intention (i.e., intention to report an error) is less likely to emerge for self-checkout machines and AI checkout compared with human checkout. The non-human nature of the interaction evokes a decreased feeling of guilt and ultimately reduces moral behavior.

Accordingly, our findings make several significant theoretical and managerial contributions. First, it extends our understanding of consumers’ reactions toward technologies, machines, and AI (e.g., Brusoni & Vaccaro, 2017; Martin et al., 2019). While past research has mainly focused on anxiety toward technological developments, we uncover an important ethical dimension created by these recent changes by incorporating the notion of moral behaviors. Second, we advance the role of guilt (e.g., Motro et al., 2018; Steenhaut & Van Kenhove, 2006) as a crucial mechanism in understanding people’s actions concerning AI and machines. We show that a decline in guilt when dealing with technologies leads to more questionable moral behaviors from individuals. Simultaneously, we eliminate several alternative mechanisms, such as perceived detectability, empathy, and preventability of the mistake. Finally, the results of this research have substantive implications. Technological developments are changing the business world (e.g., Kumar et al., 2020; Shankar, 2018); thus, more research is needed to understand how customers react to those innovations. This paper extends our understanding of moral behaviors in retail and service environments. The findings lead to straightforward suggestions for retailers and service companies regarding successful ways to decrease immoral actions (e.g., shoplifting) and work on the humanization of interactions with machines.

## Theoretical Framework and Predictions

### Technological Developments in Retailing

The landscape of retailing has drastically and rapidly changed in the past decades with the development of new technologies (e.g., Internet and mobile shopping, robots, machines, and AI), disrupting the way individuals consume and interact (Bradlow et al., 2017; Grewal et al., 2018; Rafaelli et al., 2017). The emergence of those advancements leads to interesting promises for the retail and service sector; however, it comes with its share of challenges (Grewal et al., 2018). One technology that companies, such as Amazon, Apple, and Macy’s, have started to embrace is AI, which refers to “programs, algorithms, systems and machines that demonstrate intelligence” (Shankar, 2018, p. vi) and is “manifested by machines that exhibit aspects of human intelligence” (Huang & Rust, 2018, p. 155). With its ability to accurately perform tasks and goals based on external inputs, AI is revolutionizing how companies and organizations create content, make recommendations, and interact within the store (de Ruyter et al., 2018; Haenlein & Kaplan, 2019; Weber & Schütte, 2019). Robots are also promising avenues for frontline services. For the moment, robots are mainly implemented in the manufacturing and delivery of products and services (Ivanov, 2020), but we can expect their growing presence in frontline services. Robots are anticipated to take over 20 million manufacturing jobs around the globe by 2030 (BBC, 2019).

Those changes will continue to occur within the retail environment as they represent an efficient and profitable way to build consumer experience (Ivanov & Webster, 2019). Currently, self-service technologies (SSTs) are becoming increasingly prominent in retail contexts. SSTs are described as “technological interfaces that enable customers to produce a service independent of direct service employee involvement” (Meuter et al., 2000, p. 50). SSTs have several benefits for businesses, including cost reduction, efficiency, and increasing store patronage, consumer satisfaction, and perceived control (Lee & Yang, 2013; Leung & Matanda, 2013; Rosen, 2001; Yang & Klassen, 2008; Zhu et al., 2013). However, those technologies also have associated risks. Indeed, shoplifting has increased substantially, and the techniques used by shoplifters have evolved with the presence of those machines, adding costs to retailers (Dimoff, 2020; Taylor, 2016). Extant research mainly focuses on technology adoption and how to predict consumers’ usage of those technologies. However, more research is needed to understand how consumers interact with machines and how those encounters lead to different moral standards and concerns.

### Technologies, AI, and Moral Guidance

Morality regulates human behavior by guiding behavior (what to do), predictability (what will happen), and coordination (who will do). Social and moral norms impact behaviors in various aspects of human lives (Malle et al., 2015). Besides, morality plays such an essential role in human society; therefore, it is crucial to investigate how AI and the topic of morality interact (Khalil, 1993; Martin et al., 2019). Indeed, moral decisions are considered the greatest challenge for autonomous agents, such as autonomous vehicles (Gill, 2020). “Machine ethics” or “machine morality” (Deng, 2015; Malle, 2016), the new field that studies the morality of machines, has investigated questions about the degree of moral capacity that autonomous machines should possess and how morality can be implemented (Malle, 2016). Malle (2016) proposed that the moral competence of robots should accompany (a) moral cognition and affect (e.g., judgments of blame and the process of reasoning), (b) moral decision-making and action (e.g., making choices among options), and (c) moral communications (e.g., expressing moral judgments of others’ behavior) (Gill, 2020).

In the recent “Moral Machine” experiment, millions of respondents from over 200 countries indicated their preference on how an autonomous vehicle should respond in various situations involving moral dilemmas (i.e., involving two norms inconsistent with one another; Awad et al., 2018). Overall, people preferred autonomous vehicles to save more lives rather than fewer lives, humans rather than animals, and younger rather than older lives. Besides, people favored protecting people who are lawful, with higher socioeconomic status, more physically fit, and female. Individuals also expect autonomous vehicles to minimize the overall harm (e.g., sacrifice one life to save many) (Bonnefon et al., 2016).

### Evaluation of Moral Transgressions Caused by AI (vs. Human) Agents

Researchers have investigated the morality judgment for AI. For instance, Shank and DeSeanti (2018) examined how people attribute morality and mind to AI after AI’s real-world moral violations (e.g., passport photo event involving Asians registered as having eyes closed when the eyes were actually open). They were interested in questions such as whether (1) people consider a moral violation to have occurred when an AI caused a violation; (2) people’s knowledge of the AI’s algorithm increased this perception; (3) AI is perceived to be aware, intentional, justified, and responsible for the violation; and (4) people’s perception that the AI has a mind changes these morality attributions. Participants evaluated seven real-world events in which AI committed moral violations on various items, including moral wrongness, moral attributions, and mental perception. Results showed that people do consider transgressions caused by AI as moral violations as they do the ones caused by humans. AI was attributed to have moderate levels of awareness, justification, and responsibility of the outcome. The inclusion of the algorithm information increased the perception that AI has a mind, which in turn increased attributions of intentionality and wrongness to AI (vs. other entities such as programmers; Shank & DeSeanti, 2018).

Researchers have also investigated whether people tend to apply the same norms in evaluating moral actions of AI and humans (Li et al., 2016; Malle et al., 2015). For instance, Malle et al. (2015) investigated how people apply moral norms to robots and moral judgments (of permissibility, wrongness, and blame) about their behavior. Using the “trolley problem” (Thomson, 1985), they found that robots are expected to make a “utilitarian” choice (i.e., sacrifice one person to save many lives) to a greater degree than human agents. In addition, robot agents (vs. human agents) were blamed more when robot agents did not make the utilitarian choice. These studies show that people apply different morality norms to humans and robots.

Extending these findings, Shank et al. (2019) investigated the role of decision-making structures (individual decision-making vs. joint decision-making) involving moral violations by AI and human agents. They found that humans who made a mutual decision with AI were faulted less than humans who made the individual decision. Also, people attributed more permission and less fault to AIs (vs. humans) in joint decision-making structures. Gill (2020) also investigated the dilemma between self-sacrifice and other-sacrifice in the context of AI. The author examined how people resolve the moral dilemmas of protecting themselves versus others in the context of autonomous vehicles. This research showed that people tend to consider harming others (i.e., pedestrian) (vs. self) more permissible with autonomous vehicles (vs. self) as the decision agent, which was induced by people’s attribution of responsibility to autonomous vehicles. This pattern of harming others persisted for both severe and moderate harm, but it decreased when injuring multiple pedestrians or when the pedestrian was a child.

### Consumer Morality in Consumer–Agent Interactions

The literature has accumulated knowledge on important issues, such as the use of robots and AI in retailing, AI’s morality, and moral evaluation of AI versus human agents. However, a critical question in morality–AI literature that has yet to receive much attention is people’s own moral actions toward AI agents. That is, in the AI–human interactions, what factors guide people’s moral behaviors? For example, in the retail setting, when an AI–agent (service robot or automated billing machine) made a calculation mistake, how will consumers morally behave compared to a human counterpart? With the widely expanding amount of AI in retail contexts (e.g., Malle et al., 2015), investigating the rules and norms that guide people’s behavior toward AI during AI–human interactions (vs. human–machine interactions) in the marketplace is crucial.

Previous research has investigated interactions between computers and humans. First, individuals can perceive inorganic creatures, such as robots and AI agents, to have a mind (Tanibe et al., 2017). This judgment can strongly influence perceptions of moral behaviors from those agents and toward them (Bigman et al., 2019; Tanibe et al., 2017). Indeed, the concept of mind is often bonded to morality and being morally responsible. Morality judgments are often based on the mental capacities (e.g., recognizing the consequences of actions) of the agents (Monroe & Malle, 2017). Previous research has demonstrated that people can attribute mind perceptions to robots and other technologies (Tanibe et al., 2017). Also, a stream of research theorized computers as social actors, indicating individuals’ reaction to machines according to social psychological principles (Brave et al., 2005; Nass et al., 1995; Reeves & Nass, 1996). This demonstrates the reproduction of certain behaviors in human–computer contacts and the validity of examining peoples’ reactions to machines. Still, those interactions and responses cannot be directly compared with human–human interactions (Shank, 2013, 2014). Thus, existing literature suggests that the human mind is unique, and that machines and AI cannot hold all the human mental abilities (Searle, 1980). This research stream has stipulated that we cannot equally apply human norms and moral reactions to machines (Malle, 2016; Malle et al., 2019).

An important concept to explain individuals’ differences in the interactions and responses to machines/robots is the notion of moral patiency. Indeed, exchanges with machines and robots vary in degrees of agency and patiency. Moral patiency relates to “whether someone can be the recipient of good or evil and is therefore worthy of protection” (Bigman & Gray, 2018, p. 23). Contradictory views about the moral treatments of machines and AI suggest that moral relations with those agents are not equivalent to human interactions (Bryson, 2010; Gunkel, 2018; Levy, 2009).

Another important factor that can explain those differences is along the two distinct dimensions of mind: agency and experience (Gray et al., 2017). Agency relates to the ability to do and plan, whereas experience is the facility to feel and perceive. Although humans have more agency than robots/AI agents, the main distinction revolves around the ability to experience emotions (Gray & Wegner, 2012; Gray et al., 2017). All those factors align with the notion that individuals interact with machines similarly to how they interact with humans, but they are not expected to display and obey the same social norms. Based on this research, this paper suggests a novel proposition that people will behave less morally toward technological agents than toward human agents. Thus, we hypothesize that individuals perceived interactions with machines differently and that moral intentions will be higher in human–human interactions.

In addition, machines can be perceived to exhibit more humanlike qualities and anthropomorphic cues, and intelligence induce more humanlike interactions. Previous research has found that machines, robots, and AI agents appearing more humanlike often become more appealing and generate positive feelings. Even adding minimal social cues, such as a distinctive voice or a name, can prompt humans to use social principles and paradigms (Nass et al., 1993). Thus, the more similar a machine looks to a human, the more it is perceived to have a mind. This anthropomorphism process drives individuals’ perceptions of more humanlike machines to have more moral responsibility (De Visser et al., 2016; Waytz et al., 2014). This aspect can explain why AI machines would be perceived as more humanlike than non-AI machines because they often possess more humanlike characteristics (e.g., voices, bodies, and intelligence), leading to an enhanced tendency for morality (Bigman et al., 2019). Individuals will apply and transpose moral norms and standards if they perceive AI to have more humanlike characteristics. Thus, we make the following hypotheses:

#### H1

Intention for moral behavior in human–machine interaction is lower than that in human–human interaction.

#### H2

In human–machine interactions, moral intention increases as people consider the machine more humanlike. Therefore, moral intention is higher in AI agent (humanlike machine) versus self-checkout machine agent (non-humanlike machine) conditions.

The literature has pointed out that people perceive AI to behave toward humans in a utilitarian and economical way (Li et al., 2016; Malle et al., 2015). Thus, we propose that people will perceive the relationship between AI and humans to be an exchange (rather than communal) relationship (Aggarwal, 2004; Clark & Mills, 1979; Clark et al., 1987; Yi et al., 2018). We argue that people will behave much less morally toward AI agents (i.e., AI–human relationship) compared with human agents (i.e., human–human relationship). We further propose that the decline in guilt mediates people’s decreased morality toward AI.

As mentioned, AI–human relationship is perceived as an exchange relationship; therefore, the lack of positive behaviors (i.e., acting morally) is perceived as norms rather than misbehavior. However, in the human–human relationship, in which the norms of behaviors would be more communal than those in the AI–human relationship, the lack of positive behaviors would mean a violation of the norms and thus involve guilty feelings (Yi et al., 2018). Supporting our propositions, previous research found that people are even willing to make self-harming choices to benefit the feelings of others with whom they form a communal relationship, whereas such altruistic behavior was not found for others with whom people had an exchange relationship (Yi et al., 2018).

The findings of the extant research further suggest the role of guilt in inducing the morality effect. Our research suggests a novel proposition that people will behave less morally toward AI agents than human agents. Individuals’ moral decisions are strongly embedded in emotions (Gray et al., 2017). Guilt represents one core emotion associated with moral and ethical behaviors because it is likely to impact future behaviors (Marks & Mayo, 1991). Self-conscious emotions, including guilt, relate to negative self-assessments (Baumeister et al., 1994). Guilt is often experienced when individuals perceive responsibility for transgressions or not respecting their own moral standards (Kugler & Jones, 1992; Tangney & Dearing, 2012). It can be classified into different types such as anticipatory, existential, and reactive guilt (Rawlings, 1970).

Anticipatory guilt normally stimulates ethical behaviors in consumption and retailing contexts since individuals can experience guilt when considering the possible violation of their moral standards (Kim et al., 2012; Rawlings, 1970; Steenhaut & Van Kenhove, 2005; Strutton et al., 1994). Indeed, guilt has been shown to be the critical emotion leading to more ethical, altruistic, and reparative prosocial behaviors and higher support of social causes (Deem & Ramsey, 2016; Kandaurova & Lee, 2019). The Norm Activation model is often used to explain ethical, charitable, and environmentally friendly behaviors and actions. This model states that anticipated guilt or pride will lead individuals to act in a way that is congruent with their personal norms and standards (Schwartz, 1977). This anticipation of guilt often results in avoidance behaviors or actions (Peloza et al., 2013) to avoid the negative feeling of guilt. The avoidance of possible transgressions can be linked to the Negative State relief model that stipulates individuals’ motivation to reduce negative moods (Cialdini & Kenrick, 1976). Guilt is often examined in a relationship context, and previous research has demonstrated that communal relationships can be linked to this negative feeling. Relationship contexts and norms can explain how guilt comes across because communal relationships are more about each other’s welfare, care, and commitments toward the other entity (Baumeister et al., 1994). People care more about the victim in a communal relationship; thus, guilt will often be more activated in a communal relationship (Baumeister et al., 1994). Thus, we hypothesize the following:

#### H3

Perceived guilt from not behaving morally mediates H1 and H2.

## Methodology

The above hypotheses are tested in three experimental studies to be reported. In all the studies, a moral decision scenario involving whether to report a billing error at the checkout is employed, as such situation is prevalent in retail store environments. Then, participants’ moral intentions (i.e., intentions to report the billing error) are compared between human agent and humanlike AI agent conditions. In addition, to extend our experimental setting to reflect the existing retailing environment, one study adds a new, “non-humanlike machine agent” condition (i.e., “self-checkout machine” condition). Study 1 confirms the hypothesized effect of human agent versus AI agent on participants’ moral intentions and the mediating role of “perceived guilt” of not reporting the error. Study 2 additionally employs a “self-checkout machine agent” condition to compare between humanlike versus non-humanlike machine agents (i.e., AI agent vs. self-checkout machine agent). The study further confirms the mediating role of perceived guilt across three different agents (i.e., human vs. AI vs. machine) and also evaluates (and successfully eliminates) several alternative mediators such as perceived detectability and empathy. Finally, Study 3 further examines (and finds support for) the role of “human-likeness” of AI agent.

### Study 1

Study 1 provides initial evidence of different moral responses across two checkout agent types (i.e., human vs. AI agent). Specifically, the study shows that the moral intention to report the billing error is lower in the AI (vs. human) condition, as predicted in H1 and that the effect is mediated by the perceived guilt, as predicted in H3.

#### Method: Participants, Design, and Procedure

Participants were 128 adults (*M*_*age*_ = 40.20, *SD* = 13.51; 43.8% female) recruited from Amazon Mechanical Turk (MTurk) for a nominal payment. Participants were exposed to one of two checkout agent types (human vs. AI) conditions of a between-subjects design. Based on the scenarios of Bateman and Valentine (2010), all participants were asked to imagine that they were at a local restaurant. Upon receiving a calculated bill, they found that there was a miscalculation in their favor. Then, we manipulated the agent condition (see Appendix 1 for the detailed information). Participants in the human agent condition were asked to assume they received the erroneous bill from a human waitress, whereas those in the AI agent condition were informed that they received the erroneous electronic bill from the AI system. Then, all participants were asked to indicate their moral intention to report the billing error on a 7-point scale (1 = *I definitely would take advantage of the miscalculation*, 7 = *I definitely would NOT take advantage of the miscalculation*) used in Bateman and Valentine (2010). Participants were then asked to assess their perceived guilt for not reporting the error, with three items adapted from Cotte et al. (2005) (I would feel *irresponsible/guilty/accountable* if I do not report the billing error) on 7-point scales (1 = *strongly disagree*, 7 = *strongly agree*, α = 0.884). Finally, participants’ humanlike perception of the checkout agent was measured with three items of 7-point scales that were used in Young and Monroe (2019) (1 = *mindless/unthinking/unconscious*, 7 = *mindful/thinking/conscious*, α = 0.910).

#### Results and Discussion

First, the manipulation check was successful: The waitress was perceived higher than AI in terms of human-likeness (*M*_*_human*_ = 4.09, SD = 1.51 vs. *M*_*_AI*_ = 3.24, SD = 1.88; *F* (1, 126) = 8.05, *p* = 0.005, *η*^*2*^ = 0.060). Second, as consistent with H1, participants’ moral behavior (i.e., intention to report the billing error) was higher in the human agent than in the AI agent condition (*M*_*_human*_ = 5.00, SD = 2.23 vs. *M*_*_AI*_ = 4.12, SD = 2.37; *F* (1, 126) = 4.69, *p* = 0.032, *η*^*2*^ = 0.036). Finally, participants’ perceived guilt for not reporting the billing error was greater in the human agent versus AI agent condition (*M*_*_human*_ = 5.50, *SD* = 1.72 vs. *M *_*_machine*_ = 4.70, *SD* = 2.06; *F*(1, 126) = 5.69, *p* = 0.019, *η*^*2*^ = 0.043).

To examine the hypothesized mediation mechanism in H3 [i.e., IV (checkout agent type) → Mediator (perceived guilt) → DV (moral intention)], we conducted a mediation analysis using Hayes’ (2017) Process Macro (Model 4 with 5,000 bootstrapping). The results indicated that the indirect effect via perceived guilt was significant [effect =  − 0.35, 95% CI (− 0.664, − 0.054)], whereas the direct effect became insignificant [effect =  − 0.09, 95% CI (− 0.376, 0.196)]. Thus, the hypothesized mediation was confirmed. Study 1 provided initial support for our hypotheses. 

### Study 2

Study 2 extended the results of Study 1 in several ways. First, we used a different shopping scenario that involved a greater incentive for consumers not to behave morally for generalizability of our findings. Second, we added a machine-checkout condition to the human and AI conditions of Study 1 to contrast humanlike vs. less humanlike machines. We expected that individuals would have higher intentions to morally behave in more humanlike machine agent (i.e., AI) versus less humanlike machine agent (self-checkout machines) conditions (H2). In addition, we employed a more extensive measurement of perceived guilt for the mediation test, as to be described below. Finally, we evaluated several alternative mediators of the effect (i.e., perceived detectability, empathy, preventability, accidental mistake, and honest mistake).

#### Method: Participants, Design, and Procedure

Participants were 303 adults (M_*age*_ = 33.25, *SD* = 9.82; 33.0% female) from Prolific who participated in exchange for a nominal payment. Participants were assigned to one of the three (checkout agent type: human vs. AI vs. machine) conditions of a between-subjects experimental design. We manipulated the experimental factors by using cashiers (human), automated intelligence (AI), or self-checkout (machine) as the main method for checkout. Then, all participants were asked to read a scenario describing that after leaving the store, they found that one of the purchased items was priced at $16.90, but they were only billed $1.69 (see Appendix 2 for the detailed information).

Subsequently, participants indicated their intention to report the billing error to the store on three 7-point scales (1 = *very unlikely/not probable/very uncertain*, 7 = *very likely/very probable/very certain*, α = 0.927). After that, participants were asked to indicate their perceived guilt for not reporting the billing error, using ten items adapted from Steenhaut and Van Kenhove (2006) (e.g., “I would feel tension if I *did not report* the billing error,” α = 0.955). Finally, several alternative mediators were assessed. That is, participants were asked to indicate the perceived detectability (i.e., the probability of the store being able to detect the billing error later and correct it) using three 7-point scales by LaMothe and Bobek (2020) (1 = *very impossible/very low/very unlikely*, 7 = *very possible/very high/very likely*, α = 0.910). In addition, questions about the preventability of the mistake, empathy toward the agent, and how accidental and honest (genuine) this mistake was, were also asked (see Appendix 3 for detailed measures). Finally, participants completed attention check measures, cultural orientation measures based on Triandis and Gelfland (1998), and demographic questions (including age, gender, education, ethnicity, and household income).

#### Results and Discussion

**Fig. 1:**
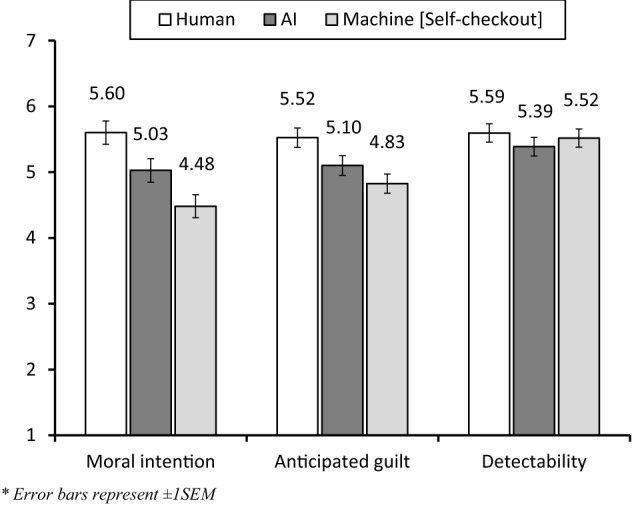
Results of study 2

For the moral intention of reporting the billing error, we conducted an ANOVA and found a significant main effect of our experimental factor [*F* (2, 300) = 10.07, *p* < 0.001, *η*^*2*^ = 0.063] (Fig. 1). Specifically, participants in the human condition (*M*_*_human*_ = 5.60, SD = 1.37) showed higher moral intention to report billing error than those in the machine condition (*M *_*_machine*_ = 4.48, SD = 2.12; LSD planned contrast *p* < 0.001) and those in the AI condition (*M*_*_AI*_ = 5.03, SD = 1.76; planned contrast *p* = 0.023). The difference between AI and self-checkout machines was also significant (planned contrast *p* = 0.03), supporting hypothesis 2, stating that moral intentions increase for more humanlike machines.

The guilt for not reporting the billing error shows a similar pattern [*F* (2, 300) = 5.77 *p* = 0.003, *η*^*2*^ = 0.037], as presented in Fig. 1. Specifically, participants in the human condition (*M*_*_human*_ = 5.52, SD = 1.26) showed higher guilt than those in the machine condition (*M *_*_machine*_ = 4.83, SD = 1.75; planned contrast *p* = 0.001) and those in the AI condition (*M*_*_AI*_ = 5.10, *SD* = 1.37; planned contrast *p* = 0.044), thus supporting H3. In addition, participants showed higher level of guilt when dealing with AI checkout compared to self-checkout machines, but the difference was not significant (planned contrast *p* = 0.187).

The perceived detectability [*F* (2, 300) = 0.56, *p* = 0.575, *η*^*2*^ = 0.004], preventability [*F* (2, 300) = 1.05, *p* = 0.351, *η*^*2*^ = 0.007], accidental mistake [*F* (2, 300) = 1.47, *p* = 0.23, *η*^*2*^ = 0.01], honest mistake [*F* (2, 300) = 0.95, *p* = 0.387, *η*^*2*^ = 0.006], and empathy [*F* (2, 300) = 1.43, *p* = 0.24, *η*^*2*^ = 0.004] were not significantly different across three experimental conditions, which cast doubts on the role of mediators of these perceptions. In addition, cultural orientation did not moderate the relationship between the different checkout types and guilt [effect = 1.01, *p* = 0.31, 95% CI: (− 0.187, 0.579)].

To examine the hypothesized mediation of perceived guilt [i.e., IV (checkout types) → Mediator (guilt) → DV (moral intention)], we conducted a mediation analysis using Hayes’ (2017) Process Macro (Model 4 with 5000 bootstrapping). Since we have three experimental conditions, we conducted three mediation analyses. First, results of mediation comparing human and machine agent conditions confirmed a significant indirect effect of perceived guilt [effect =  − 0.27, 95% CI: (− 0.468, − 0.094)]. Further, a mediation analysis comparing the human and AI conditions yields also a significant indirect effect [effect =  − 0.32, 95% CI: (− 0.631, − 0.009)]. These results confirmed H3. Finally, a comparison between AI and machine agent showed a no mediation result [effect =  − 0.22, 95% CI: (− 0.596, 0.162)], suggesting that the difference between two machine conditions (compared to the difference between human and machine conditions) was not salient in terms of the role of guilt in moral intention. This is why we will investigate more closely the perceptions of humanlike machines in section “Study 3” to demonstrate that individuals experience more guilt when they perceived a machine to be more humanlike.

These results indicate the significant mediation role played by guilt in moral intentions concerning the different checkout agents. People perceived a greater guilt for not reporting an error in a human agent versus the other two (AI and machine) conditions, and this difference drives a difference in their intentions to report the billing error.

Study 2 extended the results of Study 1 by replicating the effect AI versus human agent using a different shopping scenario, as consistent with H1. In addition, the study additionally employed a non-humanlike machine checkout (a self-checkout machine) and provided evidence for H2. Moreover, Study 2 further confirmed the hypothesized mediation of perceived guilt. In addition, other possible alternative mediators such as perceived detectability, empathy, preventability, accidental mistake, and honest mistake were ruled out. Finally, the effects were replicated with a different online sample (Prolific), which further added to the robustness of our findings.^1^

### Study 3

Studies 1 and 2 confirmed our predictions, except for a non-significant mediation result between machine and AI agent conditions. The present study remedied this limitation by providing stronger process evidence using a process-by-moderation approach. That is, Study 3 assessed individuals’ perception of human-likeness of AI in general and then examined whether the individual difference in the perception led to a difference in their moral behaviors within the context of AI checkout agent. If our assumption is correct, the humanlike perception of AI should significantly affect participants’ moral intentions. Specifically, we predicted that the moral intention is higher as participants’ humanlike perception increased.

#### Method: Participants, Design, and Procedure

Participants were 147 adults (*M*_*age*_ = 35.11, SD = 10.96; 42.2% female) recruited from MTurk in exchange for a nominal payment. First, participants were given the definition of AI from the Oxford dictionary (i.e*., the theory and development of computer systems able to perform tasks normally requiring human intelligence, such as visual perception, speech recognition, decision-making, and translation between languages*). They were then asked to express their perception of AI along a machine–human continuum on a 101-point slider bar (0 = *AI is very similar to machine*, 100 = *AI is very similar to human*). After this, all participants were assigned to the AI checkout condition of Study 2. Subsequently, participants indicated their intention to report the billing error (α = 0.947), perceived guilt (α = 0.943), and perceived detectability (α = 0.937), assessed with the same scales used in Study 2 (see Appendix 2 for the detailed information).

#### Results and Discussion

First, for the moral intention of reporting billing error, we conducted a regression analysis [IV: AI perception (a higher number represents a greater humanlike perception); DV: intention to report the billing error]. The impact of the perception of AI as human (vs. machine) on the moral intention was positive and significant (*R*^*2*^ = 0.045, *F* (1, 145) = 7.04, *β* = 0.215, *p* = 0.009), thus supporting H2. Second, a similar effect was found in the analysis of perceived guilt for not reporting the billing error (*R*^*2*^ = 0.046, *F* (1, 145) = 7.06, *β* = 0.215, *p* = 0.009), as consistent with H3. On the other hand, the perceived detectability was only marginally significant (*R*^*2*^ = 0.020, *F* (1, 145) = 2.90, *β* = 0.140, *p* = 0.091).

To evaluate the mediating role of perceived guilt (and perceived detectability), we conducted a mediation analysis [i.e., IV (AI perception) → Mediators (#1: perceived guilt and #2: perceived detectability) → DV (moral intention)] using Hayes’ (2017) Process Macro (Model 4 with 5000 bootstrapping). The results indicated that the indirect effect of perceived guilt was significant [effect = 0.156, 95% CI: (0.033, 0.281)], whereas the indirect effect of the perceived detectability was not [effect = 0.019, 95% CI: (− 0.004, 0.063)], thus further supporting H3.

This study proposes that perception of AI is critical to enhancing moral intentions of individuals. By examining the effect of humanlike perceptions of AI, we suggest ways to reduce the negative outcomes of human–machine interactions.

## General Discussion

With the omnipresence of technological developments, including the increasing presence of AI and self-service technologies in the retail environment, businesses and retailers are determined to find ways to improve the successful adoption of those technologies. Extant research confirms that those technologies can assist in reducing operating costs and lead to positive outcomes for consumers by increasing their efficiency and competency (Bulmer et al., 2018). This is especially true when those advancements are perceived as place and time convenient, thereby promoting satisfaction and store patronage (Lee & Yang, 2013; Leung & Matanda, 2013; Rosen, 2001; Yang & Klassen, 2008). However, recent data suggest that those innovations can increase undesirable behaviors from consumers, implying that individuals perceive their interactions with machines and AI differently from their interactions with humans. Previous research has examined how individuals respond morally to actions and decisions by machines and AI agents. However, consumers’ moral decisions toward those technologies are not well understood.

The present research demonstrates that individuals’ different perceptions of social and moral standards in their interactions with humans versus technologies. Our three studies address this element by examining moral behaviors in a retail environment. These three experiments provided empirical evidence that people have lower moral intentions toward technologies (i.e., machines and AI) than toward humans (hypothesis 1). Intentions to engage in moral behavior (i.e., reporting a billing error) are lower when dealing with a technology (i.e., machine or AI) versus human, especially for less humanlike machines (i.e., self-service checkouts). In addition, Study 3 indicated that more humanlike perceptions of machines will significantly impact moral intentions (hypothesis 2). Indeed, regarding AI as human leads to a higher likelihood of reporting a billing error compared with more machine conceptions. Finally, the studies establish that perceived guilt mediates this relationship. When dealing with another human being or a technology they recognize as more human, individuals will project more guilt related to a possible violation of their moral standards (hypothesis 3).

### Theoretical Contributions

Our research makes several important theoretical contributions. It extends the literature on technological developments and AI in the morality domain by showing that consumers interact differently with those technologies and that the interactions can lead to questionable moral behaviors from individuals. The existing literature has mainly focused on examining technological benefits and issues related to AI and identifying factors to optimize the adoption level of those technologies (De Bruyn et al., 2020; Lee, 2004). Recent research has also focused on the moral judgments and responsibility of robots and AI technologies (Bigman et al., 2019). Indeed, a lot of recent research has been focusing on how to assure the creation of ethical machines, ensuring that machines act in accordance with a set of ethical principles. Researchers have concentrated their efforts on understanding how individuals perceive morality for machines and how to increase moral judgments from available technologies (Serafimova, 2020). However, people’s responses to machines seem to be the opposite, as individuals are less concerned with moral behaviors when interacting with technology. Negative unethical outcomes related to those technologies have been observed in the business world, requiring attention. Although some companies inappropriately use AI, the lack of human interactions has also caused questionable moral actions from the consumers.

Examining this aspect, we uncovered that a checkout involving a technology (i.e., machines and AI) reduces the intentions to engage in moral behaviors. This suggests that people perceive their interactions and behaviors with technological advancements to be different from human interactions and that the moral concerns and reactions in those situations vary. This result extends our understanding of machines as social agents, indicating that even though individuals apply social principles during their interactions with technologies, they do not replicate the same level of social and moral norms. By examining the effect of humanlike perceptions, we present suggestions on how we can reduce this negative effect from people. In study 3, we propose that perception of AI is critical to enhancing moral intentions.

Another novel contribution of this research is that it directly examined one of the affective mechanisms responsible for the effect of technologies on moral judgment. Specially, we show that perceiving technology as more human might enhance moral behaviors. Hence, when consumers perceived AI as more human, moral concerns may become more influential, resulting in stronger moral intentions to report an error, whereas a machine perception may lead to less ethical concerns. Besides, one important contribution of this research is to demonstrate the importance of the expected guilt in explaining how human interactions encourage moral actions. Indeed, when dealing with a human or a technology perceived as more human, people expect more guilt when thinking about a potential transgression of their moral standards. Indeed, previous exploratory research suggested that the decreased likelihood of getting caught might increase theft (University of Leicester, 2016), our research indicates that perceived detectability doesn’t drive the effect, but the projected feeling of guilt.

### Managerial Implications

Practically speaking, our findings can help understand how to maximize the benefits of technologies, including AI, without increasing the unaccountability of individuals that can lead to questionable moral behaviors. First, AI and technological advancements are used to improve the efficiency and personalization of the consumer experience. The extant research shows how the adoption of these innovations can be increased, whereas our findings suggest that construing AI as more human represents an additional aspect to consider in undermining the potential risks of interacting with those machines and ensuring the same level of moral standards and concerns from individuals. Additionally, COVID-19 is expected to increase our reliance on new technology (Kim et al., 2021), meaning that our results can have a more substantial impact on corporations and business owners.

Second, according to the findings of this study, guilt is an essential factor in moral intentions. Indeed, guilt mediates the relationship between the type of interaction and moral intentions. This should be examined by marketers and retailers when developing and managing AI technologies. Drawing from our results, we recommend businesses and retailers to consider factors in the representation of those technologies that can embody higher humanization. Such anthropomorphism techniques should increase the level of perceived guilt and thus motivate people to adopt moral actions.

Importantly, managers must avoid the uncanny valley. Indeed, this relationship states that individuals can experience a feeling of unease or strangeness when robots/machines appear too humanlike (Ho & MacDorman, 2017; Mori et al., 2012). Practitioners must ensure that they create AI agents with abilities that fit with their appearance. For example, robots that look more humanlike also need to have more humanlike mental capacities and companies must avoid bad character animations. Another important issue to consider is what makes a machine/AI more humanlike. Anthropomorphizing (e.g., voice, face, and name) has been used successfully to increase human perceptions, but other factors can play a crucial role. Mental abilities of machines and AI agents to elicit actions (i.e., agentic mind) and the capacity for feelings and emotions (i.e., experiential mind) could help develop moral intentions toward technologies. More research is necessary to understand the exact roles of those capacities concerning moral actions.

### Limitations and Directions for Future Research

We recognize that our studies have certain limitations that offer avenues for future research. First, this study was administered through online panels providing experimental scenarios portraying potential situations. Although hypothetical scenarios have been used in the past (e.g., Kim et al., 2021), including AI checkout, further research needs to be conducted to replicate these results in real interactions with technologies. Moreover, although the use of online panels such as Amazon Mechanical Turk and Prolific can be an effective method (e.g., Crump et al., 2013), future studies should replicate the effects with participants in a more controlled setting.

Second, our studies used a generic manipulation of AI. Future studies should employ more realistic manipulations of AI. With the rise and importance of AI for both practitioners and researchers, more online experiments will continue to include manipulations of AI and AI agents (e.g., Aoki, 2021; Awad et al., 2018).

Third, even though we showed significant mediation results for Study 3, the conclusion was based on correlation rather than causal relation since we measured AI perception. Future research must manipulate the perception of AI to provide a strong causal relationship. In addition, our research only involves moral intentions and concerns. Future studies can investigate whether those moral concerns and intentions do transpire into actual behaviors and feelings of guilt. Also, past research has demonstrated that temporal distance (Agerstöm & Björklund, 2009) and motivations (e.g., economic gain; LaMothe & Bobek, 2020) can influence moral concerns. Examining these gaps in relation to technologies and retail settings represents interesting avenues for future research.

In addition, a deeper understanding of the different factors of a relationship affecting this effect can be important avenues for future research. First, examining the type of consumer-organization relationship can have a significant impact. Communal and long-term relationships can be perceived to be more important for consumers, thus influencing their moral intentions toward the firm. Also, an interesting avenue for future investigation would be to examine different firm sizes (e.g., small retailer vs. big corporation). The size of the business could impact reactions; drawing from the prevailing B2B literature, we found that smaller firms (or retailers) typically exercise more risk with their stakeholders (and partner organizations) and rely more heavily on a larger cross-section of customers/clients (Croonen, 2010). In a more B2B-specific context, future research can examine how the power differences between the different actors influence the trust dynamics between retailers and customers. Besides, in this context, inter-organizational trust is frequently maintained and executed via individuals, or boundary spanners, acting on behalf of their respective organizations that may enjoy higher levels of relationship quality with their customers/clients. A potential moderating factor within that of a more industrial-type marketing relationship of exchange (buyer–supplier relationship) as those individuals (e.g., sales representatives or key account managers or other boundary spanners) are most typically responsible for representing their own organization in the relationship and, consequently, may affect the type of reporting behavior we are investigating.

Future research should also examine contextual factors that could influence our results. For example, people’s perception of privacy can be systematically differences between people from Eastern or Western cultures in terms of perceiving morality and behaving morally. Even though we measured culture orientation in study 2 and did not find any interaction effect with our manipulations, we can think that culture could influence elements related to retailing morality) (e.g., privacy perception differences between Eastern and Western cultures).

Finally, people might have a different perception of familiarity with AI-based checkout. Previous research has indicated the tendency for people to show less morality in less familiar (vs. familiar) situations. For example, people showed a lower level of morality in a foreign language (Costa et al., 2014). Therefore, the higher morality in human (AI) condition could be explained by the difference in familiarity between human (familiar) and AI (less familiar). However, if familiarity is the underlying mechanism, the moral intention of reporting the billing error should also differ between the AI and machine conditions as AI is less familiar than the machines; however, no significant difference was found (Study 2). Thus, the familiarity-based explanation is unlikely to be viable. Nevertheless, further investigation for the possible role of familiarity would be desirable.

## Appendix 1: Stimuli of Study 1

### Scenario for Human Condition

Imagine that you are at a local restaurant. The waitress has just given you the calculated bill for your meal. Upon looking at your bill, you notice that the waitress has miscalculated it in your favor. In this situation, what do you do?

### Scenario for AI Condition

Imagine that you are at a local restaurant. The smart checkout system, operated by artificial intelligence (AI), has just given you the electronic bill for your meal. Upon looking at your bill, you notice that the AI system has miscalculated it in your favor. In this situation, what do you do?

### Perceived Guilt Measurement

I would feel irresponsible if I don’t report the billing error.

I would feel guilty if I don’t report the billing error.

I would feel accountable for not reporting the billing error.

(1 = *strongly disagree*, 7 = *strongly agree*).

### Manipulation Check

To what extent do you think the following traits are descriptive for *the waitress (AI)* in the above scenario?

(1 = *mindless/unthinking/unconscious*, 7 = *mindful/thinking/conscious*).

## Appendix 2: Stimuli of Studies 2 and 3

### Scenario for Human Condition

The store uses cashiers as the main method for check out.

Right after leaving the store, you checked to see if you had all of your belongings with you and to verify your receipt. You found out that one of the items you purchased was priced at $16.90, but you were only billed $1.69.

Apparently, there must have been some sort of billing error.

### Scenario for AI Condition

The store uses checkout-free store operated by automated artificial intelligence.

Right after leaving the store, you checked to see if you had all of your belongings with you and to verify your receipt through your smartphone. You found out that one of the items you purchased was priced at $16.90, but you were only billed $1.69.

Apparently, there must have been some sort of billing error.

### Scenario for Machine Condition (Study 2 only)

The store uses self-checkout machines as the main method for check out.

Right after leaving the store, you checked to see if you had all of your belongings with you and to verify your receipt. You found out that one of the items you purchased was priced at $16.90, but you were only billed $1.69.

Apparently, there must have been some sort of billing error.

### AI Information and AI Perception (Study 3 only)

Do you know what Artificial Intelligence (AI) is?

AI can be defined as *the theory and development of computer systems able to perform tasks normally requiring human intelligence, such as visual perception, speech recognition, decision-making, and translation between languages.*

## Appendix 3: Additional Measurements of Study 2

Anticipated guilt measurement (Steenhaut & Van Kenhove, 2006):

1. I would feel tension.
2. I would feel remorse.
3. I would think that I was in the wrong.
4. I would think that I shouldn’t have done what I did.
5. I would feel like undoing what I have done.
6. I would feel like punishing myself.
7. I would apologize.
8. I would avoid meeting people’s gaze.
9. I would want to make up for what I have done wrong.
10. I would want to be forgiven.

(1 = *strongly disagree, 7* = *strongly agree*).

### Perceived Detectability Measurement

How would you estimate the probability of the store to detect this billing error and correct this error in future? (1 = *very impossible,* 7 = *very possible*).

How would you estimate the probability of the store to detect this billing error and correct this error in future? (1 = *very low,* 7 = *very high*).

How would you estimate the probability of the store to detect this billing error and correct this error in future? (1 = *very unlikely,* 7 = *very likely*).

### Preventability Measurement

How preventable was the mistake? (1 = *not at all preventable,* 7 = *highly preventable*).

### Accidental Mistake Measurement

Was it an accidental mistake? (1 = *not at all accidental*, 7 = *very much accidental*).

### Honest Mistake Measurement

Was it an honest mistake? (1 = *not at all honest,* 7 = *very much honest*).

### Empathy Measurement

I try to look at everybody’s side of a disagreement before I make a decision.

In this situation, I usually try to ‘‘put myself in their shoes.”

I would describe myself as a pretty soft-hearted person.

(1 = *strongly disagree,* 7 = *strongly agree*).

## Footnotes

1 In an earlier version of a similar study, we found similar effects with participants recruited from Amazon MTurk (*n* = 259 adults (*F* (2, 256) = 3.46, *p* = .032, *η*^*2*^ = .026), showing the difference among the three conditions (*M*_*_human*_ = 4.42, SD = 2.17 vs. *M*_*_machine*_ = 3.55, SD = 2.15 vs. *M*_*_AI*_ = 4.06, SD = 2.22). The study also supported the mediating role of guilt on the moral intention of reporting the billing error and refuted the possible role of perceived detectability. The detailed results can be accessed from the authors.
